# Angiodysplasia in Renal Disease Patients: Analysis of Risk Factors and Approach to Manage Such Patients

**DOI:** 10.7759/cureus.9784

**Published:** 2020-08-16

**Authors:** Sadhika Verma, Marline A Attallah, Maria Daniela Jarrin Jara, Avneesh S Gautam, Safeera Khan

**Affiliations:** 1 Internal Medicine, California Institute of Behavorial Neurosciences and Psychology, Fairfield, USA; 2 Internal Medicine, California Institute of Behavorial Neurosciences and Psychology, Fairfiled, USA

**Keywords:** angiodysplasia, renal disease

## Abstract

Gastrointestinal bleeding due to angiodysplasia is a common problem in patients with renal insufficiency. There are several theories to explain the increased occurrence of these lesions in this specific group of patients, including various metabolic factors and existence of comorbidities. Advancements made in diagnostic measures have helped route the approach in patients with different risk factors and have also helped solve the dual purpose involving therapeutic intervention with endoscopy. We conducted a thorough literature search on PubMed to extract relevant data. A total of 29 articles were chosen after applying the inclusion and exclusion criteria. Although the clinical presentations may vary in this cohort of patients, and bleeding is known to stop spontaneously, a conservative approach may not be enough. Endoscopic treatment, use of hormones like estrogen, octreotide, and vasopressin, arterial embolization, and lastly surgery are valuable therapeutic tools.

## Introduction and background

Gastrointestinal (GI) angiodysplasia is a frequently encountered problem in patients with renal disease. It is also the most frequent cause of recurrent bleeding in patients with renal failure [1]. The prevalence of angiodysplasia in renal failure patients ranges from 19% to 32% compared to only 5% in individuals with normal renal function [1,2]. Angiodysplasia presents at a younger age in patients than in the general population. Angiodysplasia is a vascular malformation with a diameter of less than 1 cm within the mucosal and submucosal layer of the bowel [1]. Multiple hemorrhagic episodes are frequent and mostly occult and intermittent [1,2].

Clinical features of angiodysplasia consist of iron-deficiency anemia, hemoccult-positive stool, and melena, and in some cases, hematemesis. Hemorrhage is usually painless, and the bleeding ceases spontaneously in at least 90% of the cases [1,3]. Angiodysplastic lesions can be present in any part of the GI tract and are usually more than 1 in number [3,4]. Although the exact etiology of angiodysplasia is still unknown, several studies suggest a degenerative vascular process accelerated by the hypo-oxygenation of the intestinal mucosa secondary to atherosclerotic peripheral vascular disease or other underlying condition, like in this study, renal failure. Intermittent submucosal venous obstruction and intermittent arterial flow lead to the degenerative process. Several contributory factors accelerate this process, such as uremic platelet dysfunction, dialysis, use of non-steroidal anti-inflammatory drugs (NSAIDs) and antiplatelet drugs, impaired calcium metabolism, constipation, and increasing age.

Diagnosis can be made by various endoscopic methods, such as capsule endoscopy, colonoscopy, and enteroscopy. However, the efficacy of other modalities like technetium-99m (99mTc) scintigraphy has also shown promise. The first and the most effective therapeutic option for angiodysplasia is endoscopic coagulation. However, treatment options may vary depending on the patient factors. The use of octreotide, conjugated estrogen, and thalidomide has also proven to be effective.

We aim to enhance our understanding of the approach used to manage a renal disease patient with angiodysplasia and how various factors determine the use of different modalities for diagnosis and treatment in such a patient.

## Review

Method

Study literature was searched manually on PubMed using regular keywords for data collection. The keywords were 'Angiodysplasia' and 'Renal disease', using which we found a total of 121 articles. We selected articles from the database since the inception of the topic to date. We included studies that were in English and focused on human subjects only. Finally, we selected 20 articles from PubMed for review. We also extracted some articles that were relevant to the topic and satisfied the inclusion criteria, from within the references of the selected studies.

The search results for regular keywords are shown in Table 1.

**Table 1:** Regular Keywords for Literature Search and the Number of Studies

Keywords used	Database used	Number of studies screened	Number of studies selected
Angiodysplasia	PubMed	2,300	1,483
Angiodysplasia and renal disease	PubMed	121	88

Inclusion/Exclusion Criteria

We used the following Inclusion criteria for our data search, screening, and extracting studies for review. We only selected studies involving human subjects and were published in the English language only. We searched for relevant observational studies, clinical trials, randomized controlled trials, literature reviews, and systematic reviews as our selected studies for review paper.

While screening and extracting papers, the animal studies and the papers that were in languages other than English were removed. Meta-analyses, books, documents, and gray literature were also excluded.

Results

**Figure 1:**
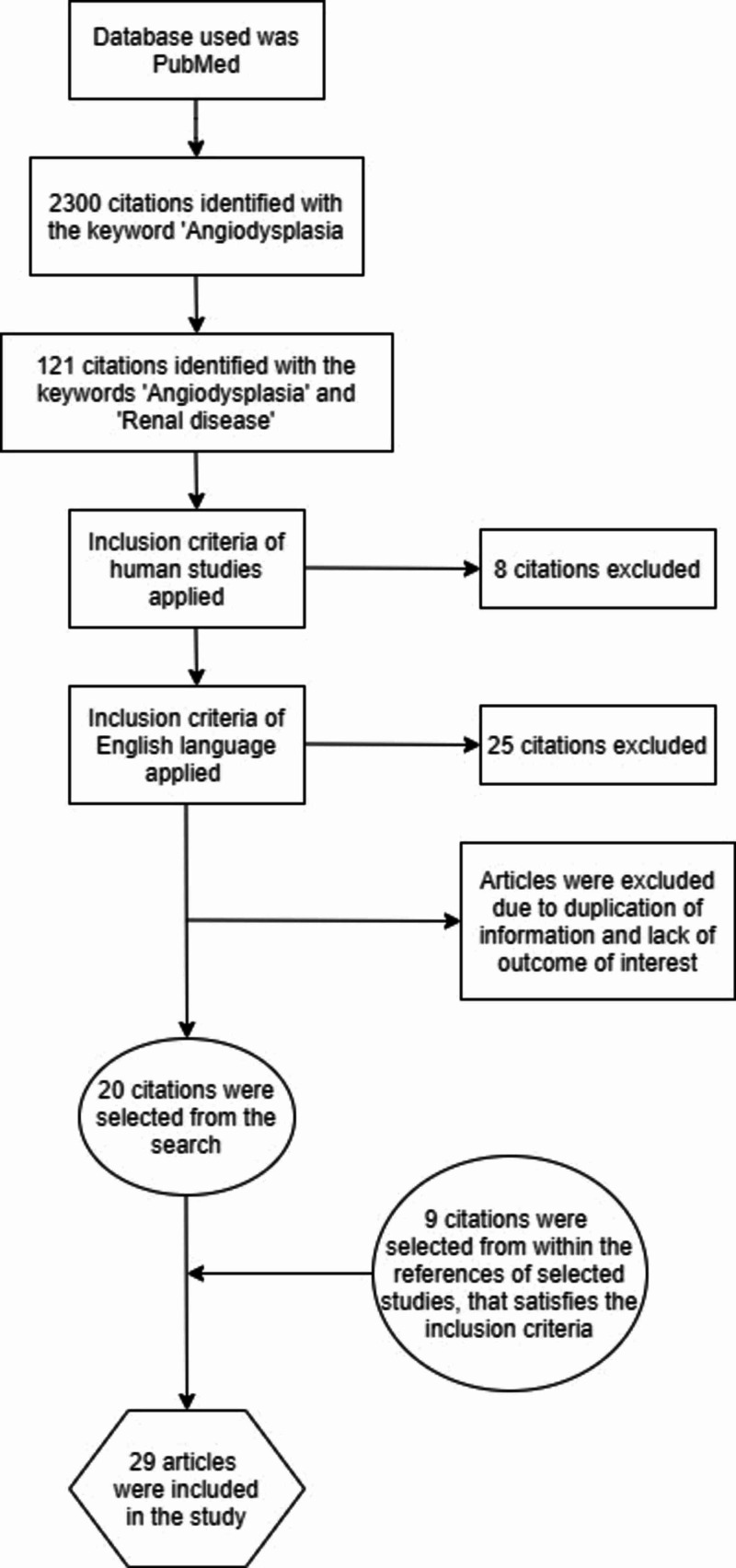
PRISMA Flow Diagram Depicting the Process of this Literature Review PRISMA, Preferred Reporting Items for Systematic Reviews and Meta-Analyses

We included a total of 29 studies to review. These studies included two systematic reviews, three non-randomized controlled clinical trials, and three randomized controlled trials. Ten out of these were observational studies, five of them were case series, five were case reports, and one of them was a review article. The total number of subjects, including all of the studies, were 11,156. The PRISMA (Preferred Reporting Items for Systematic Reviews and Meta-Analyses) flow diagram showing the process of this literature review can be seen in Figure 1.

Discussion

Angiodysplasias are intestinal vascular malformations, red in color, flat, or slightly elevated on the mucus lining with a diameter between 2 and 10 mm [1]. GI bleeding due to angiodysplastic lesions is a problem frequently seen in patients with renal disease, irrespective of dialysis. In individuals with normal renal function, the prevalence of angiodysplasia as a cause of bleeding is 5%, as compared to 19% to 32% in patients with chronic renal failure. It is also indicated as one of the most common causes of recurrent bleeding in such patients [1]. According to the most recent United States Renal Data System (USRDS) database, the prevalence of chronic kidney disease (CKD) was 15% and that of end-stage renal diesase (ESRD) was 2160.7 per million among the United States populations. GI bleeding is a major cause of morbidity and mortality in renal disease patients [2].

Clinical Presentation of Angiodysplasia

Clinically, angiodysplasia may present as an un­explained iron-deficiency anemia, hemoccult-positive stool, and melena or hematochezia. The bleeding can be chronic or acute. It is usually painless and ceases spontaneously in most of the cases, but is often recurrent. About half of the patients with angiodysplasia have more than one lesion, and lesions can be located in the stomach, small intestine, or even colon [3]. A retrospective analysis conducted by Mai et al. indicated the association of rebleeding due to angiodysplasia with the multiplicity of the lesions, hence leading to increased hospitalization rates [4].

Microscopically, angiodysplasia is seen as dilated and distorted, thin-walled vessels, lined by endothelium, and in some cases by a thin layer of smooth muscle. Endoscopically, angiodysplasia lesions are 2-10 mm in size, flat or slightly raised above the mucosal surface, and che­rry red in color [3]. Angiodysplasia usually presents in patients older than 60 years in the general population, while in patients with CKD, they may be seen at a younger age [1]. Angiodysplasia can be asymptomatic as well. A retrospective study by Kim et al. also revealed strong associations of bleeding with the size of the lesions (larger than 1 cm) [5].

When located in the stomach, gastric antral vascular ectasia (GAVE), or the so-called GAVE syndrome, is given a common name 'Watermelon stomach'. It is characterized by the appearance of parallel longitudinal red columns along mucosal folds, as a result of dilated and thrombosed capillaries within submucosa and mucosa, along with fibromuscular hyperplasia in lamina propria and thickening of the submucosa with the dilated tortuous venous channel [6].

Etiopathogenesis and Associated Risk Factors

The etiology of angiodysplasia and its association with CKD are still not well known. However, there have been several theories that attempt to understand pathogenesis. Angiodysplasia seems to be a degenerative vascular process secondary to intermittent submucosal changes. The intramural components of the colonic blood vessels are prone to chronic, intermittent, and partial obstruction due to motility of the intestines, especially at the places where the vessels penetrate the muscular layers of the colon wall. Chronically persistent and repeated trauma to the vessel wall can lead to malformations and, eventually, arteriovenous communications. Hypoxia of the intestinal wall can be exacerbated due to several comorbidities, such as atherosclerotic peripheral vascular disease and diabetic microangiopathy that can affect the microcirculation of the colon, further limiting the blood supply and, subsequently, mucosal oxygenation [7].

Several other factors, such as aging, uremic platelet dysfunction, use of NSAIDs and antiplatelet drugs, and cardiovascular comorbidities, may contribute to the development of angiodysplasia lesions [5,7].

Another possible correlation can be made with hypercalcemia as a result of hyperparathyroidism in CKD patients, which can cause intestinal vascular calcifications that eventually decrease their permeability, compromising the microcirculation of the intestinal mucosa, causing hypoxia, and, the vulnerability of the vessels to repeated trauma [7,8].

Most of the theories proposed about the pathogenesis of angiodysplasia have a close relationship with each other, like constipation. Constipation is a frequent problem in CKD patients and can be attributed to hypercalcemia, reduced fiber intake, use of hemodialysis, administration of phosphate binders, and presence of diabetes. Constipation can cause an increase in the intraluminal pressure of the large bowel, causing distension. According to the Laplace law, the wall tension is proportional to the intraluminal pressure and the radius. The increased wall tension can exacerbate mucosal and submucosal hypoxia [7].

A case study done by Nambiar et al. focused on the relation of the use of sevelamer in CKD patients and mucosal defects. Hyperphosphatemia is a common occurrence in CKD patients, and sevelamer is a phosphate binder often used in its treatment. Sevelamer is well known to be associated with the deposition of its crystals in the GI tract, vascular calcification, and endothelial damage. Coincidental sevelamer deposition on a previously injured area along with foreign body reaction observed strongly suggests casualty [9].

Riva et al. studied a patient in which they observed improvement of angiodysplasia bleeding after a switch of peritoneal dialysis (PD) to hemodialysis. This can again be explained by La Place's law, as the PD solution load increases peritoneal pressure, subsequently increasing wall pressure and contributing to pathogenesis. The study also stated that angiodysplasia should be considered as a relative contraindication to PD, because angiodysplasia treatment can cause peritonitis in patients receiving PD [10].

The various proposed theories for angiodysplastic bleeding are shown in Table 2.

**Table 2:** The Various Proposed Theories for Angiodysplastic Bleeding YOP, year of publication; DM: diabetes mellitus; NSAIDs: non-steroidal anti-inflammatory drugs; PD; peritoneal dialysis; CKD: chronic renal failure

Study	Journal	YOP	Study type	Conclusion
Matesanz et al. [8]	Nephron	1987	Case series	Progressive aging of the patient, cardiovascular comorbidities, and defective calcium-phosphorous metabolism can attribute to formation of angiodysplastic lesions.
Galanopoulos [7]	Saudi Journal of Kidney Diseases and Transplantation	2012	Review	Angiodysplasia seems to have a vascular degenerative process presumably due to repeated trauma, causing intermittent submucosal venous obstruction and hypoxemia. Cardiovascular comorbidities and DM aid this process. Other contributory factors are hypercalcemia, constipation, uremic platelet dysfunction, and use of antiplatelet drugs and anitcoagulants.
Kim et al. [5]	Korean Journal of Internal Medicine	2016	Observational study	Bleeding due to angiodysplasia can be linked to uremic platelet defects and abnormal function of von Willebrand factor. History of DM and use of NSAIDs or aspirin may also be contributory.
Riva et al. [10]	Peritoneal Dialysis International	2016	Case report	Laplace's law can explain how PD solution load can increase peritoneal pressure and intestinal wall pressure and in turn cause bleeding, especially in the right colon. Use of anitcoagulants in hemodialysis can also contribute to bleeding.
Nambiar et al. [9]	Case Reports in Nephrology	2018	Case report	Use of sevelamer to treat hyperphoshatemia in CKD patients can cause endothelial damage and vascular calcifications in the intestinal wall, and hence can cause bleeding.

Other GI Abnormalities in Renal Disease Patients

GI bleeding is a frequently tackled cause of morbidity and mortality in renal disease patients. A cross-sectional study conducted by Agudo et al. focused on endoscopic abnormalities that can be observed in renal disease patients like, esophagitis, acute lesion of the gastric mucosa (ALGM), chronic gastritis, preneoplastic lesions (e.g., colonic polyps), diverticulosis, inflammatory bowel disease, hemorrhoids, and angiodysplasia [11]. Other lesions that can be observed are inflammatory gastric polyps, duodenal erosions, and nodular duodenum. Among these, gastric and duodenal ulcerations remain among the most frequent causes of bleeding [12].

Other Associations of Angiodysplasia

Angiodysplasia is known to be associated with various clinical entities, such as chronic renal insufficiency, aortic stenosis (Heyde's syndrome), von Willebrand disease, cirrhosis, and many autoimmune disorders (primary biliary cirrhosis, diabetes, scleroderma) [2,6].

**Figure 2:**
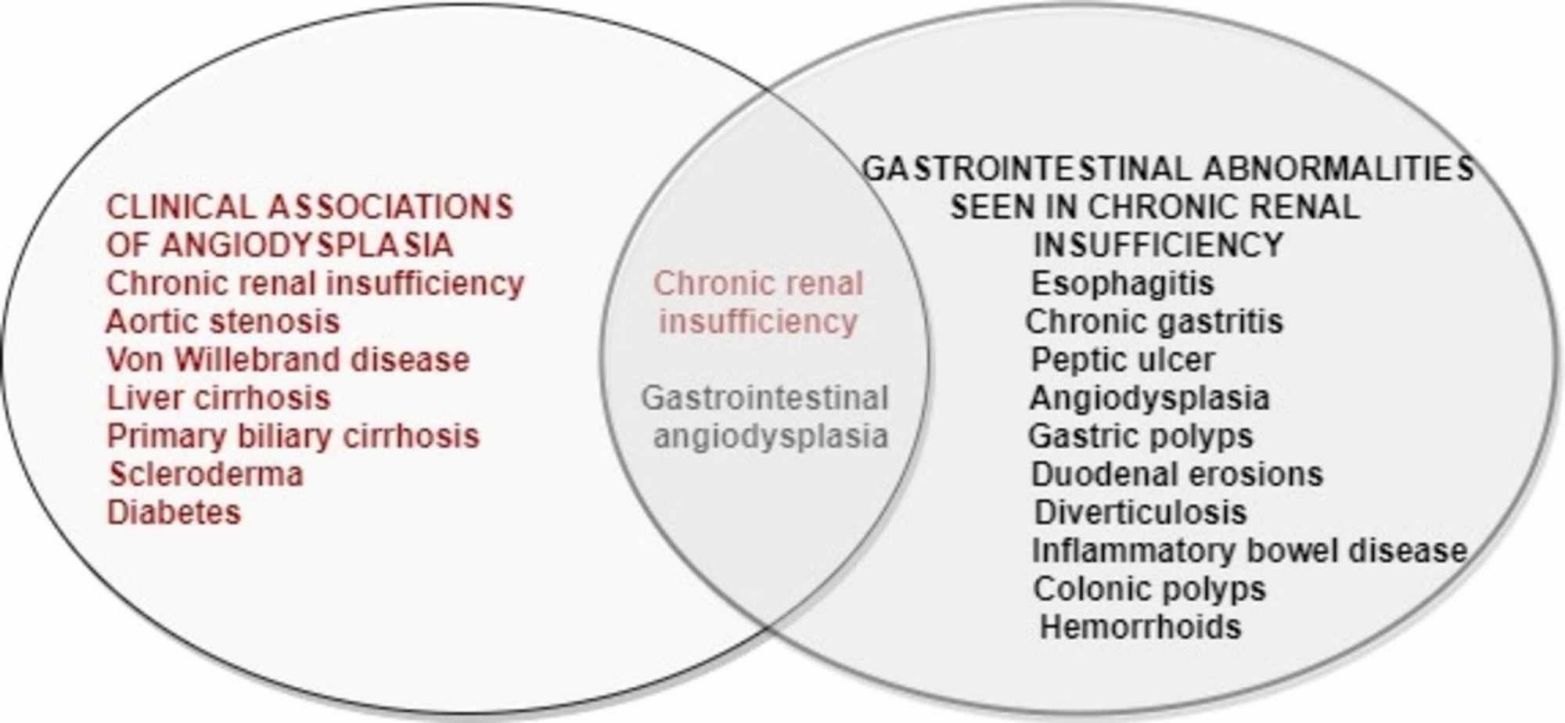
The Association and Correlation of Renal Disease and Angiodysplasia

With this understanding, the association and correlation of renal disease and angiodysplasia can be seen in Figure 2.

Diagnosis

Many diagnostic modalities have gained importance. Endoscopy is usually the first step in the approach towards diagnosing a patient with angiodysplasia. Traditionally, esophagogastroduodenoscopy (EGD) and colonoscopy can be used depending on the suspected location of the lesion.

Wireless capsular endoscopy (WCE) has become the investigation of choice in such patients [13]. A randomized prospective study by Leung et al. indicated that WCE has a higher diagnostic yield and better long-term outcomes when compared to angiography [14].

Video capsule endoscopy (VCE) is an effective means of diagnosis and has a higher yield when done soon after the onset of bleeding. The characteristics of a lesion on VCE may be used to evaluate the bleeding tendency of that lesion and can be used for an appropriate selection of patients for double-balloon endoscopy (DBE) [15,16].

Device-assisted enteroscopy (DAE) is a technique that includes DBE, single-balloon enteroscopy, through-the-scope balloon-assisted enteroscopy, and spiral enteroscopy. These techniques are used for a thorough evaluation of the small bowel and also allow for therapeutic intervention [16,17].

DBE is a newly developed device useful for identifying the source of intestinal bleeding as well as a therapeutic tool for performing non-surgical hemostasis for bleeding in the small intestine. It can be inserted more deeply into the small intestine via either an oral or anal approach. The combination of capsule endoscopy (CE) and DBE can be a complementary approach for renal disease patients with intestinal bleeding [16,18].

Push enteroscopy is a useful technique with a higher yield than CE, especially in patients with suspected proximal small bowel lesions. It may be a better alternative in patients with recurrent bleeding [16].

CT angiography (CTA) has shown to have a high diagnostic yield when done in a patient with ongoing GI bleeding or immediately after the bleed and also allows a better understanding of the therapeutic intervention. However, the use of contrast limits the use of CTA in patients with renal insufficiency [19].

Red blood cell (RBC) scintigraphy is an effective imaging technique used in identifying lesions causing lower GI bleeding in patients for whom other diagnostic tests have yielded no results. It is also helpful in patients with lower rates of bleeding, particularly if a patient presents later after the onset of bleeding [20,21].

Treatment

Endoscopic coagulation: Endoscopic coagulation therapy is the mainstay of treatment in patients with active bleeding due to angiodysplasia. Argon plasma coagulation (APC) and bipolar coagulation can be used with low complication rates, decreased need for transfusion, and prevention of rebleeding. A case study done by Ibáñez-Sanz et al. suggested that radiofrequency ablation (RFA) using the HALO90 system can be used as a real and safe option for refractory bleeding due to GAVE after attempts of APC in patients with chronic renal failure [22].

However, many studies report a minimal difference in long-term outcomes in those treated with endoscopy vs. observation alone. In a majority of patients, bleeding ceases spontaneously [23]. Observation, along with as-needed transfusions and iron replacement, is potentially a viable option for patients for whom sedation is risky or those who have had multiple prior endoscopic interventions with recurrent or persistent bleeding. Iron replacement is done intravenously as oral iron is ineffective in renal disease patients [16].

Octreotide: Octreotide is a somatostatin analog that acts by decreasing splanchnic blood flow, inhibiting angiogenesis, improving platelet aggregation, and increasing vascular resistance [16].

It is known to be effective in patients with recurrent bleeding refractory to endoscopic interventions. Combination therapy of endoscopic coagulation and octreotide can lead to a significant reduction in the need for transfusion and rebleeding [16,24].

Conjugated estrogen: Estrogen therapy in renal disease patients who present with GI bleeding due to angiodysplasia has been effective, lowering the number of bleeding episodes as well as the need for transfusion. Although the exact mechanism of action is unknown, the use of conjugated estrogens carries advantages over other aggressive treatment methods, particularly in patients with multiple angiodysplasia lesions or high-risk comorbidities (e.g., CKD, elderly). Adverse effects are observed when it is used in higher doses, which is usually not required. Thus, estrogen is effective, especially when a longer duration of action is needed, and the immediate onset of the effect is not crucial [25,26].

Thalidomide: Thalidomide is an immunomodulating, antiangiogenic (effect on vascular endothelial growth factor [VEGF]) drug known for its notorious teratogenic effects. It can be used in angiodysplasia patients in whom endoscopic treatment has failed or cannot be used or in patients with multiples lesions [27].

A randomized study conducted by Ge et al. showed that plasma VEGF levels after the treatment were decreased significantly in patients with angiodysplasia, compared with levels before treatment. Also, the decrease in VEGF levels was more significant in responders than in non-responders [27]. However, thalidomide carries a risk of severe adverse effects, such as deep vein thrombosis, and common adverse effects, such as fatigue, constipation, dizziness, peripheral neuropathy, and peripheral edema.

Desmopressin: The mechanism of action is multifactorial. It causes an increase in the release of von Willebrand factor (vWF) and factor VIII (FVIII) complexes, improved platelet membrane receptor binding of the vWF: FVIII complexes, and direct action on the platelet membrane leading to increased platelet serotonin uptake and subsequent adenosine triphosphate release. The same effect of desmopressin on platelet function is also achieved by hemodialysis. It is relatively shorter acting and reported to be effective in preventing bleeding when used before surgical procedures or endoscopic therapy [16].

A crossover clinical trial conducted by Malyszko et al. indicated that the use of desmopressin shortens the prolonged bleeding time when given in uremic patients [28]. However, the effect of desmopressin on transfusion requirements and its effectiveness in preventing GI bleeding have not been studied well.

Arterial embolization: Selective embolization with decreased complication rate has been made possible with advances in technology [16].

Surgery: Surgery is usually reserved for patients with refractory and life-threatening cases of bleeding. The primary surgical intervention is intraoperative enteroscopy, followed by surgical resection or endoscopic therapy. Surgery is also effective in the lysis of adhesions that would prevent the use of advanced endoscopic procedures [16].

Since endoscopic procedures for small-bowel evaluation usually have a high negative predictive value, there is minimal use of intraoperative enteroscopy in diagnosis. It is typically reserved for patients who have a lesion already detected by other endoscopic modalities and need further localization during surgery before an intervention [16].

Tranexamic acid: It may be used in patients in whom interventional procedures cannot be done [29]. However, its efficacy and use concerning various patient factors have not been studied well. 

The different diagnostic and therapeutic modalities are mentioned in Table 3.

**Table 3:** The Different Diagnostic and Therapeutic Techniques Studied Over the Years YOP: year of publication; RCT: randomized controlled trial; 99mTc, technetium-99m; RBC: red blood cell; CRF: chronic renal failure; VEGF: vascular endothelial growth factor; APC: argon plasma coagulation; CKD: chronic kidney disease; EGD: esophagogastroduodenoscopy; VCE: video capsule endoscopy; DBE: double balloon enteroscopy; CTA: CT angiography

Study	Journal	YOP	Study type	Conclusion
Livio et al. [26]	The New England Journal of Medicine	1986	RCT	Conjugated estrogen can be used as an alternative to cryoprecipitate or desmopressin for the treatment of bleeding associated with renal failure, especially when a longer duration of action is required and immediate onset of the effect is not important. The mechanism of action of estrogen is not clear.
Malyszko et al. [28]	Folia Hematologica	1990	Clinical trial	Desmopressin shortens prolonged bleeding time in uremic patients probably thorough serotonergic mechanism.
Vujkovac et al. [29]	American Journal of Kidney Diseases	1998	Case report	Tranexamic acid was successfully used in the management of a hemodialysis patient, in whom endoscopic treatment or surgery could not be done.
Oliveras et al. [20]	Nephron	1998	Observational study (prospective)	99mTc RBC scintigraphy may be the preferred diagnostic technique for angiodysplasia, especially in patients with CRF, in whom angiography (use of contrast) may further detiorate renal function.
Manzanera et al. [25]	Nefrologia	2005	Case series	Use of estrogen in renal disease patients with angiodysplasia bleeding has been safe and effective, lowering recurrence as well as transfusion needs. Its main advantage (over sclerotherapy) is that it can act on multifocal lesions.
Karagiannis et al. [13]	World Journal of Gastroenterology	2006	Observational study (prospective)	Wireless capsule endoscopy is the diagnostic method of choice in patients with obscure gastrointestinal bleeding, and especially in patients with CRF.
Olmos et al. [23]	Diseases of Colon and Rectum	2006	Observational study (prospective)	Endoscopic argon plasma ablation therapy is an effective choice of treatment in renal disease patients with angiodysplasia, when conservative treatment alone is not enough.
Yamazaki et al. [18]	NDT Plus	2009	Case series	Double balloon endoscopy is useful in both diagnosis and treatment of intestinal bleeding in hemodialysis patients.
Brown et al. [24]	Digestive Diseases and Sciences	2010	Systematic review	Octreotide can be successfully used to treat bleeding due to angiodysplasia decreasing the need for transfusions, and high-risk interventions. It is specifically effective in patients with refractory bleeding and inaccessible lesions.
Dolezal et al. [21]	Digestion	2011	Clinical trial	RBC scinitgraphy is an effective means of detecting lesions that cause bleeding in the small intestine.
Ge et al. [27]	Gastroenterology	2011	RCT	Thalidomide, despite its adverse effects, can be effectively used in treatment of angiodysplasia. Thalidomide has an effect on VEGF levels.
Leung et al. [14]	The American Journal of Gastroenterolgy	2012	RCT	Capsule endoscopy has a higher diagnostic yield than angiography in patients with acute obscure gastrointestinal bleeding.
Fan et al. [17]	Journal of Digestive Diseases	2013	Observational study (prospective)	Balloon-assisted enteroscopy is an effective approach to treat angiodysplasia.
Rahmi et al. [15]	Endoscopy	2014	Clinical trial	Video capsule endoscopy can be used to detect lesions that cause bleeding and their bleeding potential, and for a better selection of patients for double balloon enteroscopy.
Ibáñez-Sanz et al. [22]	Revista Española de Enfermedades Digestivas	2015	Case report	Radiofrequency ablation using the HALO90 system can be an effective option in renal disease patients with refractory bleeding due to angiodysplasia, in whom APC has been tried.
Tseng et al. [19]	PLoS One	2015	Observational study (retrospective)	CTA is effective technique to identify lesions causing acute, overt, obscure gastrointestinal bleeding. But its use is limited in patients with renal diseases due to risk of contrast nephropathy.
Muftah et al. [16]	Annals of Gastroenterology	2019	Systematic review	In patients with CKD, obscure gastrointestinal bleeding can be diagnosed using tools like EGD, VCE, DBE, CTA, and RBC scintigraphy. Besides conversative management, other therapeutic options are endoscopic coagulation, octreotide, desmopressin, thalidomide, arterial emobolization, and surgery.

Limitations

There were not enough clinical trials assessing the use and efficacy of several diagnostic and therapeutic modalities that are available for a renal disease patient presenting with angiodysplasia. Therapeutic options other than endoscopic interventions need to be studied further to provide broader coverage of the patient group.

## Conclusions

Angiodysplasia, being a common cause of GI bleeding in renal disease patients and one of the most common reasons for recurrent bleeding in the same group of patients, emphasizes the need to study the risk factors and the pathogenesis involved in its development. Our review focused on understanding the risk group and the various diagnostic and therapeutic methods that are indicated in such a patient. The etiopathogenesis is still not clear. However, it seems to be related to the vulnerability of the walls of the intestines to hypoxic changes, exaggerated by uremic changes. Advancements made in endoscopic procedures have helped in both the diagnosis and treatment of this group of patients. This condition can be managed conservatively, or if needed, through endoscopic coagulation, embolization, and surgery. There is still a need to explore other options as a potential and alternate treatment options like octreotide, estrogen, and thalidomide, since not all patients are suitable candidates for traditional methods.
